# Cascaded processing enables continuous upstream processing with *E. coli* BL21(DE3)

**DOI:** 10.1038/s41598-021-90899-9

**Published:** 2021-06-01

**Authors:** Stefan Kittler, Christoph Slouka, Andreas Pell, Roman Lamplot, Mihail Besleaga, Sarah Ablasser, Christoph Herwig, Oliver Spadiut, Julian Kopp

**Affiliations:** 1 Research Division Integrated Bioprocess Development, Institute of Chemical, Environmental and Bioscience Engineering, TU Wien, Gumpendorfer Straße 1a, 1060 Vienna, Austria; 2 Institute of Chemical, Environmental and Bioscience Engineering, TU Wien, Getreidemarkt 9/166, 1060 Vienna, Austria

**Keywords:** Industrial microbiology, Microbiology techniques

## Abstract

In many industrial sectors continuous processing is already the golden standard to maximize productivity. However, when working with living cells, subpopulation formation causes instabilities in long-term cultivations. In cascaded continuous cultivation, biomass formation and recombinant protein expression can be spatially separated. This cultivation mode was found to facilitate stable protein expression using microbial hosts, however mechanistic knowledge of this cultivation strategy is scarce. In this contribution we present a method workflow to reduce workload and accelerate the establishment of stable continuous processes with *E. coli* BL21(DE3) exclusively based on bioengineering methods.

## Introduction

*Escherichia coli* is frequently employed in research and industry for the production of recombinant proteins ^1–3^. The advantages of a large toolbox for genetic modifications as well as fast doubling times outweigh the negative traits, such as the lack of posttranslational modifications ^3–6^. Lactose and the synthetic structural analogue IPTG (Isopropyl-β-d-thiogalactopyranosid) are common transcription inducers for T7-based *E. coli* expression hosts, like BL21(DE3) ^7–9^. Whereas IPTG is known to cause cellular stress ensuing toxic effects at concentrations higher than 1 mM ^8,9^, no such effects have been reported for lactose ^7,10–12^. High concentrations of recombinant product additionally have been reported of causing host cell toxicity ^13^, potentially favoring the use of lactose over IPTG due to slower heterologous protein expression. Furthermore, induction by lactose increases the production of soluble product ^6,14–16^. Recently, we reported that co-utilization of glycerol and lactose promotes recombinant protein expression and increases viability compared to a mixed-feed with glucose ^17,18^. Unlike glucose, glycerol is integrated into glycolysis and gluconeogenesis ^17,18^. Results of other studies also indicate altered TCA-activity on glycerol compared to glucose metabolism potentially favoring recombinant protein production ^19–21^.

For *E. coli* bioprocesses it is known that batch and fed-batch cultivations result in unwanted variable, time-dependent productivity ^22^, however these processing modes are still state-of-the-art for industrial applications. Time-independent processing would reduce batch to batch variations and consequently allow stable productivity and robust downstream processing ^23,24^. Furthermore, a change from fed-batch to continuous manufacturing would allow a reduction in scale and operating costs and thus should be pursued as the most efficient cultivation technique ^25–27^. However, solutions for continuous cultivation with *E. coli* have not been realized yet, since subpopulations evolve over elongated cultivation times ^13,28–30^, which are yet to be investigated ^13,31^.

Previous studies highlighted a cascaded continuous operating system using two bioreactors, to reduce subpopulation formation compared to common continuous cultivations (i.e. chemostat, turbidostat, etc.) ^13,32–34^. Even though a spatial separation of biomass formation and recombinant protein production seems promising, the effects of process parameters on productivity in such a cascaded continuous cultivation are barely known ^10,35^. Further, long-term effects beyond 100 h of induction and thus the potential for continuous manufacturing with *E. coli* have not been investigated yet ^32,34,36^ because the development of continuous processes is highly time- and resource-dependent.

In this study, we investigated the cascaded continuous cultivation with a T7-based *E. coli* host over extended cultivation times of more than 220 h using cell specific productivity as a key performance indicator to determine cell equilibrium state. Glycerol-lactose systems were utilized solely for continuous process development, due to their beneficial results over glucose-fed and IPTG-induced systems in diverse pre-studies^18,32,37^. After intensive screening and variation of selected process parameters, a workflow on how to set-up continuous cultivations with *E. coli* BL21(DE3) was developed, tested and verified. Consequently, we present a first workflow to set up and accelerate stable continuous process development with *E. coli* BL21(DE3).

## Results

**Figure 1:**
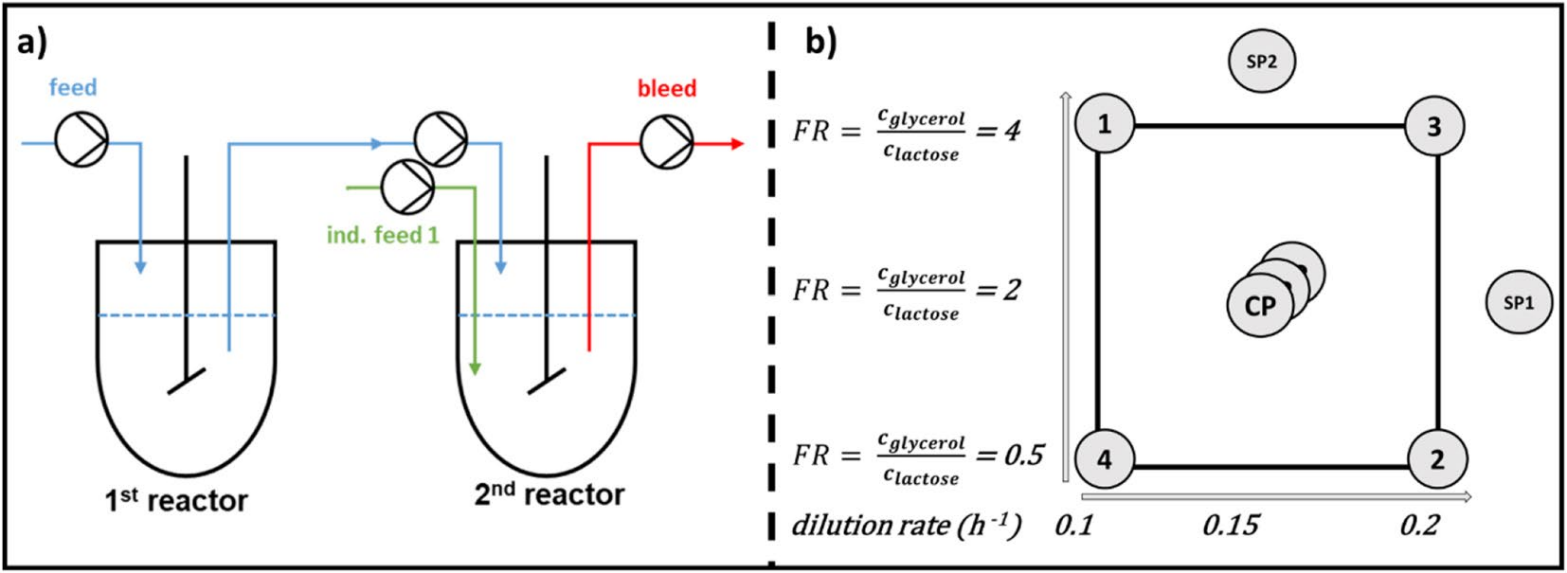
(**a**) Process overview of the cascaded continuous cultivation. Reactor 1 is used for biomass formation. Biomass stream is transferred to reactor 2, where recombinant protein production is induced; (**b**) showing the full factorial design of experiment (DoE) conducted for the factors dilution rate and feed ratio with the responses space–time yield and specific productivity, center point experiments were conducted in triplicates. (1–4 = run1– run 4, *SP* starpoint, *FR* feed ratio).

Cascaded continuous biomanufacturing with *E. coli* BL21(DE3) was set up as shown in Fig. 1a. Process performance of the cascaded reactor system was evaluated via specific productivity (q_p_ [mg/g/h]) and space time yield (STY [mg/L/h]). The STY was chosen as indicator to determine the volumetric product throughput, which is frequently used to evaluate continuous processes. The process parameters dilution rate and glycerol-lactose mixed feed ratio were varied according to a multivariate design (Fig. 1b). The design space was based on recent findings on effects of the dilution rate ^32^ and the feeding rates using glycerol ^18^ in our group. Two model proteins with completely different expression behavior were chosen as model proteins in this study: The N-Pro fusion protein which is obligatory expressed as inclusion body in *E. coli* was used for initial screening ^38^. The gained Know-how and the proposed workflow was then tested with the fluorescence protein mCherry, being predominantly expressed in soluble form ^39^.

### Prior knowledge on the E. coli host producing N-Pro

To screen the expression of recombinant proteins in a cascaded continuous operating system, we used a recombinant *E. coli* BL21(DE3) strain producing a N-Pro fusion protein. Essential physiological parameters, which are required for process development, have been determined in previous studies and are summarized in Table 1.

**Table 1:** Prior knowledge on the expression strain BL21(DE3) producing the N-Pro model protein.

Parameter	BL21(DE3) [N-Pro]	Refs.
Maximum specific growth rate—µ_max_/h	0.38	^18^
Maximum specific lactose uptake rate—q_s,lac,max_ [g/g/h]	0.23	^18^
Maximum specific productivity—q_p,max_ [mg/g/h] in fed batch	70 at a q_s,gly_ of 0.4–0.5 g/g/h	^40,41^
Induction temperature [°C]	31.5	^22^
Induction pH [−]	6.7	^22^

It is known that the dilution rate significantly affects the productivity in a cascaded cultivation system ^32,34^. As a high number of variables in a multivariate design space would result in a remarkably high experimental load, we started with univariate experiments varying the specific glycerol uptake rate q_s,gly_ during induction, by keeping the total dilution rate constant (Reactor 2; Fig. 1a). Hence, the biomass flux and the induction feed flux into reactor 2 were varied in different percentages (10–30% induction feed of total volumetric stream in reactor 2; Eq. (1)) measuring STY and q_p_ as a response. We did not test percentages higher than 30% induction feed to prevent sugar accumulation in reactor 2. The concentration of carbon source to inducer was fixed at a 2:1 ratio. This ratio was based on fed-batch results for concomitant C-source and inducer uptake ^42^ to ensure complete uptake of the inducer lactose. Based on previous findings, where stable productivity was achieved for 36 generations ^32^, we fixed the total dilution rate at 0.15/h.

1 $$D_{{{\text{screening }}}} = D_{{{\text{reactor }}1}} + D_{{{\text{mixed feed reactor }}2}} = 0.15/{\text{h}}.$$

First screening experiments showed best results when the total dilution rate in reactor 2 was composed of 30% mixed feed addition (= D_mixed feed reactor 2_ in Eq. (1)). The other 70% of the volumetric stream into reactor 2 were assembled via biomass and base addition. Details are given in Supplementary Data Sect. 1.1.

**Figure 2:**
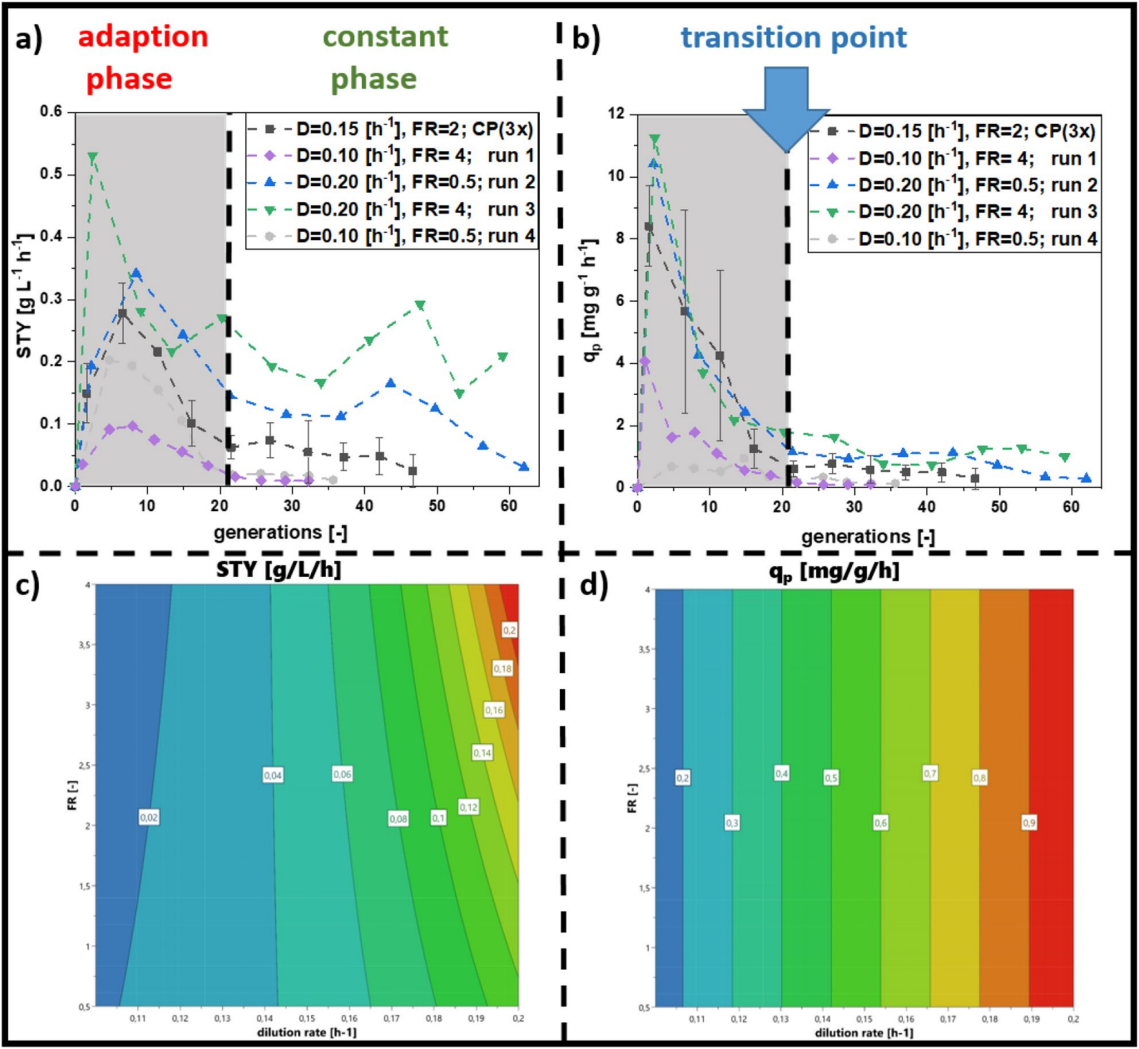
Results derived from the multivariate experiments carried out for cascaded continuous cultivation for the N-pro protein. Centerpoint runs (CP) were summed up in a trend line (triplicate). (**a**) Space–time-yield (STY), (**b**) specific productivity (q_p_), contour plot for (**c**) STY and (**d**) for q_p_. High dilution rates, as well as a high uptake rates of glycerol and low rates of lactose seem to be preferable to achieve a high specific productivity and a high STY (*FR* feed ratio, *D* dilution rate).

In the following multivariate design, we fixed the induction feed at 30% and changed the dilution rate and the induction feed ratio (Fig. 1b), which represents the influx of primary carbon source (glycerol) to inducer (lactose) ^18^. All performed cultivations followed the same trend: STY and q_p_ reached a maximum after the start of induction and then decreased after 20 generations (Fig. 2a,b). Afterwards, productivity either stabilized at a certain level or further decreased to zero. The decrease of q_p_ in the adaption phase can be explained by the adaptation time of biomass to inducing conditions. Thus, we subdivided the process into an adaptation phase until 20 generations, before a “transition point” marked the switch towards a constant phase (Fig. 2).

**Figure 3:**
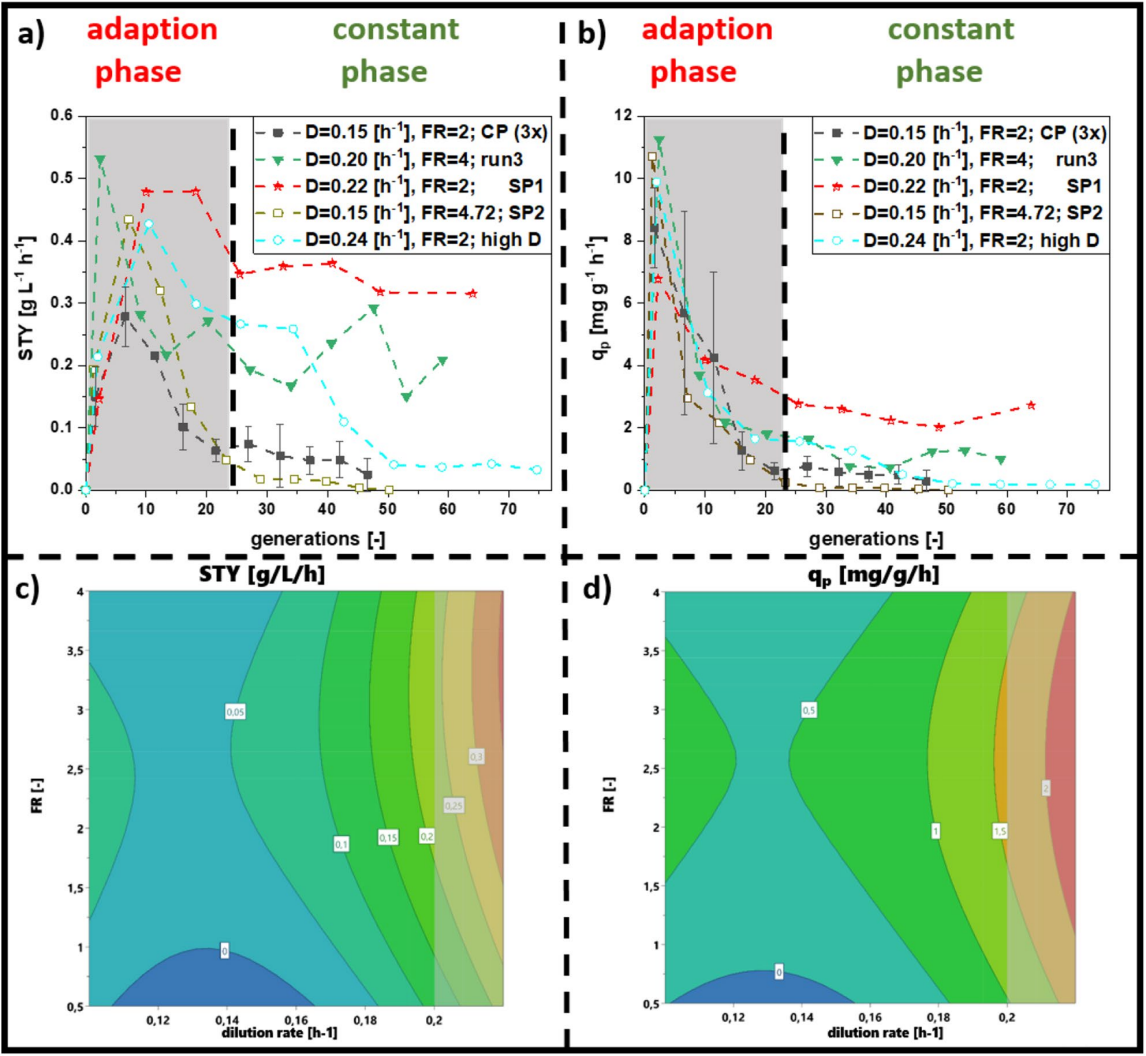
Comparing the optimum run of cascaded continuous cultivations derived from the DoE (design of experiment) (run 3) and the centerpoints, with star-point experiment 1 (D = 0.22/h with F_in,gly_ = 6.6 g/h and F_in,lac_ = 3.3 g/h), star-point experiment 2 (D = 0.15/h with F_in,gly_ = 14.25 g/h and F_in,lac_ = 3.3 g/h) and a further increase in dilution rate (D = 0.24/h with F_in,gly_ = 6.6 g/h and F_in,lac_ = 3.3 g/h) (**a**) STY (space–time-yield) (**b**) q_p_ (specific productivity): Contour plots for (**c**) STY and (**d**) q_p_ (*FR* feed ratio, *D* dilution rate, *SP* star point).

Long-term recombinant protein production was possible in numerous cultivations in the design of experiment (DoE), however STY and q_p_ varied significantly. Runs at a dilution rate of 0.2/h and the feed ratio of 4 (Supplementary Data 2.1) led to the highest STY and q_p_. However, these cultivations showed high fluctuations in STY and were not regarded as stable. The contour plots (Fig. 2c,d) visualize the results, tending towards higher dilution rates and higher feed ratio. Dilution rate showed the highest parameter impact, but also mixed feed composition influenced the model (Supplementary Data 2.2). As process conditions resulting in highest productivity were at the edge of the design space (Fig. 2c,d), we added star-point experiments with a distance of $\sqrt 2$ according to a central composite circumscribed (CCC) model, namely (1) D = 0.22/h with a feeding ratio = 2, and (2) D = 0.15/h with feeding ratio = 4.72 (Fig. 3).

The process operated at a D = 0.22/h with a feeding ratio = 2 gave a high productivity of 0.35 g/L/h even in the constant phase for a prolonged cultivation time of 250 h. At these conditions we further determined stable STY and q_p_. We included the star point experiments in the multivariate design and modeled the response in terms of STY and q_p_ (Fig. 3c,d). All model parameters are given in the Supplementary Data 2.2.

As µ_max_ for this strain was determined at 0.38/h, the known limit of a stable process was found at a dilution rate of 0.3/h considering biomass yield decrease upon recombinant protein production (Supplementary Data 1.2). To avoid wash-out of host cells, we tested yet another process condition between the new targeted optimum and 0.3/h, namely at D = 0.24/h (with an identical feed ratio of 2). Specific productivity was found stable from 60 to 80 generations throughout the process operated at D = 0.24/h. However, the process operated at star-point 1 with D = 0.22/h achieved higher stable STY and q_p_ and was thus found to be optimal. Summing up, we could identify a narrow operating space for cascaded continuous processing using the N-Pro model protein in *E. coli* BL21(DE3), which led to stable long-term protein expression. In a next step, we summarized our findings in a workflow protocol which comprises two phases.

### 2-phase workflow for setup of a cascaded continuous process for *E. coli* BL21(DE3)

#### Phase I

**Figure 4:**
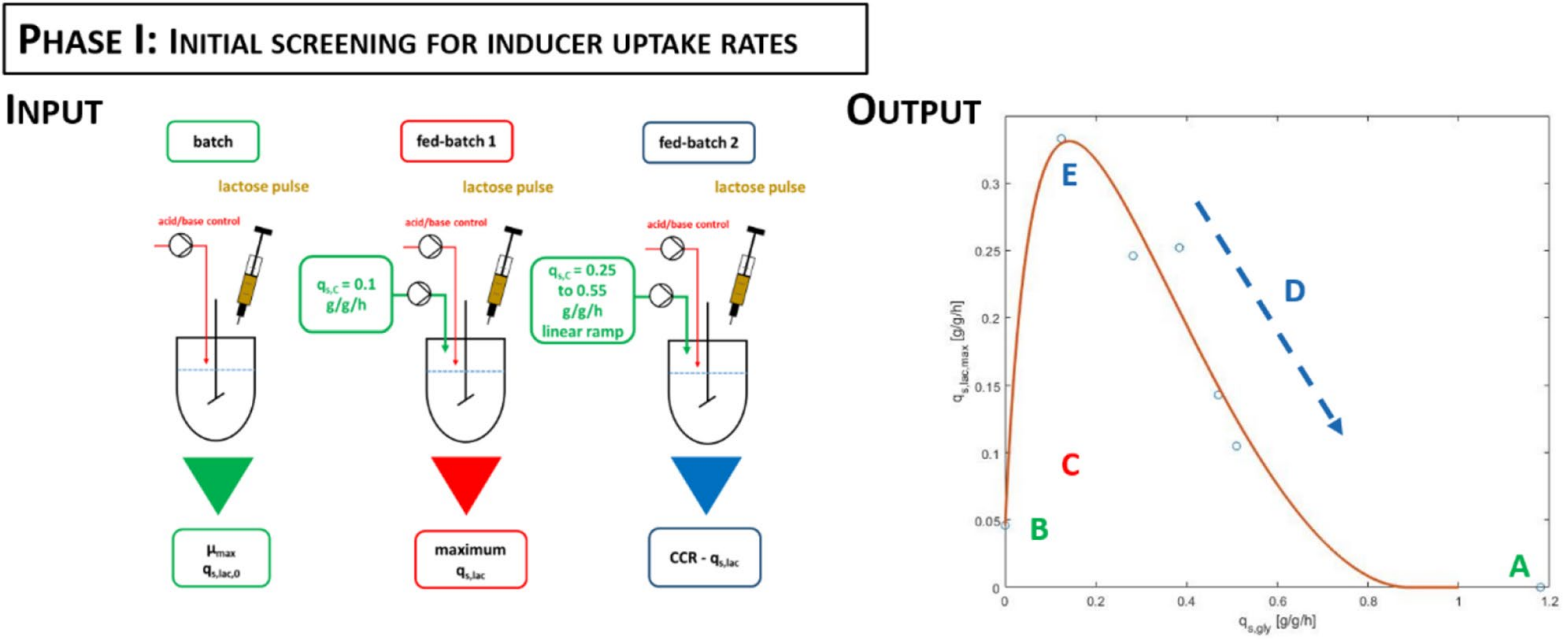
Phase I: Generic workflow for determination of maximum inducer uptake rates (q_s_) versus primary substrate uptake rates for *E. coli*. Therefore three experiments are necessary: (1st step) a batch cultivation for maximum growth rate (A) and inducer uptake without C-source (B); (2nd step) fed-batch cultivation for the maximum inducer uptake rate (C) (3rd step) fed-batch cultivation for inducer uptake rates between a q_s,gly_ set point from 0.25 to 0.55 g/g/h using a linear ramp approach to screen the regime of carbon catabolite repression (CCR) (E,D).

For the strain producing the N-pro fusion protein essential physiological data was already known from previous studies (Table 1). To determine this essential pre-knowledge, solely three bioreactor runs in a batch-/fed-batch mode without the need of sophisticated analytics (i.e. OMICs-studies) are necessary. Phase I is thus subdivided into three steps to obtain the required knowledge:

In step (I) the maximum growth rate and specific inducer uptake rates (q_s,lac_) are determined. Maximum growth rate is easily available upon a batch fermentation with excess carbon source. After the batch, lactose is pulsed in the reactor and uptake of lactose is monitored in a timely resolved way, e.g. by sampling every 30 min (Fig. 4, first reactor). The outcome of the first cultivation provides the maximum growth rate µ_max_ and the inducer uptake rate at q_s,gly_ = 0, namely q_s,lac,0_.

Furthermore, the mechanistic relation between q_s,gly_ and inducer uptake rate (q_s,lac_) must be determined. This can be done in a series of static experiments: A fixed q_s,gly_ is applied, inducer is pulsed in excess and lactose decrease in the broth is measured ^42^. However, using this approach a high number of cultivations is necessary. We recently showed that this relation can be obtained more elegantly in only three experiments using dynamic feeding during induction ^42^. Therefore, in step (II) a fed-batch is performed at q_s,gly_ of 0.1 g/g/h to determine the maximum q_s,lac_ (Fed-batch 1 in Fig. 4). In step (III) q_s,lac_ as function of q_s,gly_ can be identified using a linear q_s,gly_ ramp. Inducer is pulsed, q_s,gly_ is increased steadily from 0.25 to 0.55 g/g/h and regular samples are taken to determine q_s,lac_ in the regime of carbon catabolite repression (CCR) (Fed-batch 2, Fig. 4).

#### Phase II

As intensive screening of cascaded continuous cultivation has been conducted for the N-pro protein, we hypothesize that determined dilution rate optima for the N-pro protein can be transferred to the expression of other proteins using glycerol as C-source, reducing the experimental workload. Based on the previous findings, we recommend to alter the dilution rates in a narrow range of 0.16 to 0.22/h. In addition moderate to high uptake rates (q_s,gly_ = 0.25–0.55 g/g/h) were found to result in high recombinant protein production in fed-batches ^22,32,40–43^. Hence, we recommend to vary the feed ratio for screening near to the maximum inducer uptake rate, to prevent inducer deficiencies and also investigate moderate to high sugar uptake rates (q_s,gly_).

**Figure 5:**
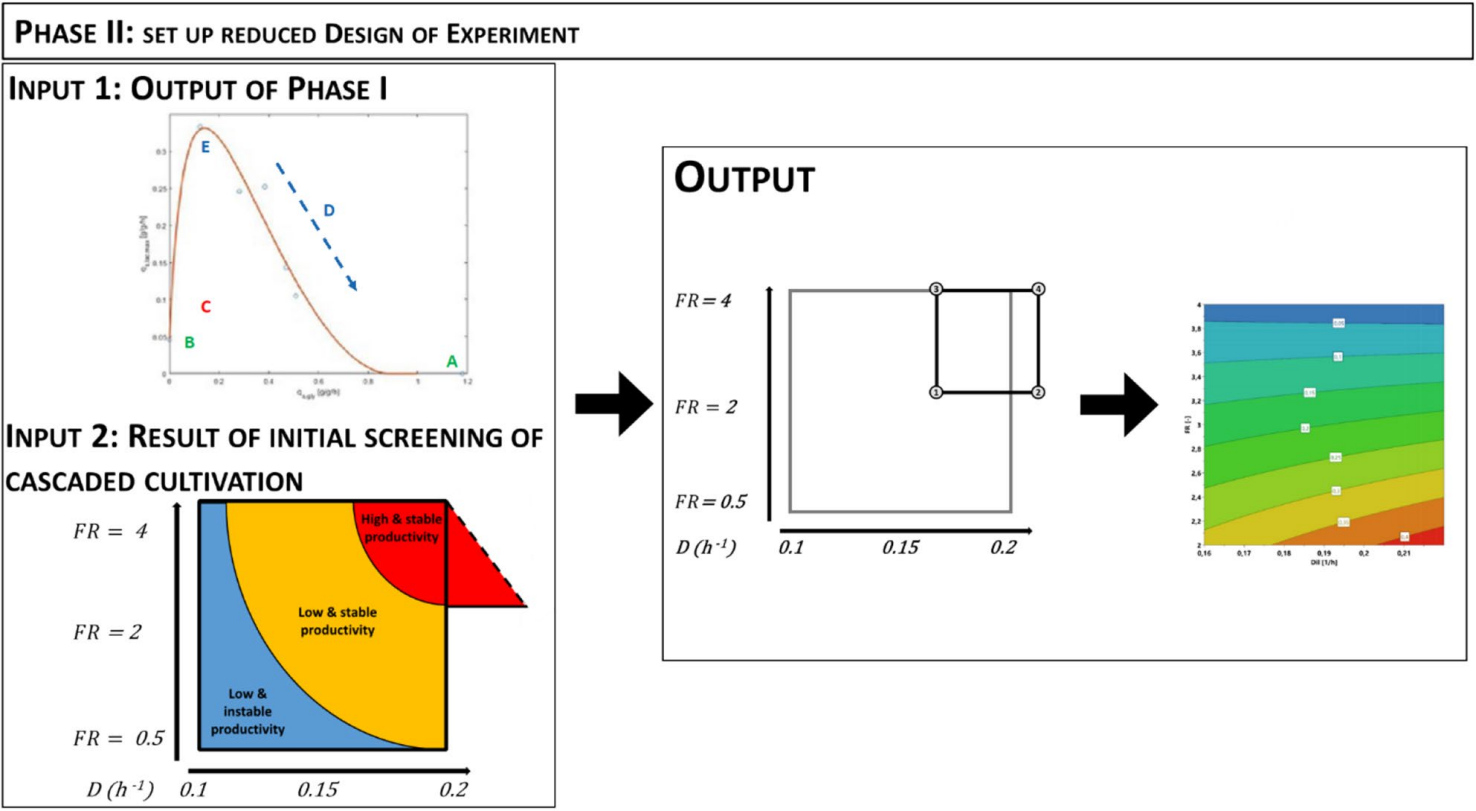
The previous findings of cascaded continuous cultivation are used and combined with the output of Phase I to choose a reduced design space. The obtained constricted design space in phase II accelerates the process to find optimal process conditions (*FR* feed ratio, *D* dilution rate).

Consequently, our strategy to establish cascaded continuous cultivations for *E. coli* BL21(DE3) using glycerol as carbon source and lactose as inducer, can be summarized as: In phase I physiological parameters are determined (Fig. 4) to estimate a design space for proper feed ratios for cascaded continuous cultivation. In phase II, the output of phase I is combined with the results obtained from initial screening to set up a reduced design of experiment (DoE) in a constricted design space (Figs. 2, 3, 5).

### Testing the proposed workflow to establish cascaded cultivation using *E. coli* BL21(DE3)

To test whether the proposed workflow shows platform character, we conducted the procedure proposed in Figs. 4 and 5 for BL21(DE3) expressing the fluorescence protein mCherry. In phase I, inducer uptake rates at varying feeding rates were determined. This allowed to compose feed ratios for the mCherry protein in cascaded continuous cultivation (Outcome of phase I; Fig. 4).

The effect of the dilution rate was already screened intensively in previous cultivations conducted with the N-Pro protein. Hence, dilution rates were altered in a narrow range of 0.16 to 0.22/h.

**Figure 6:**
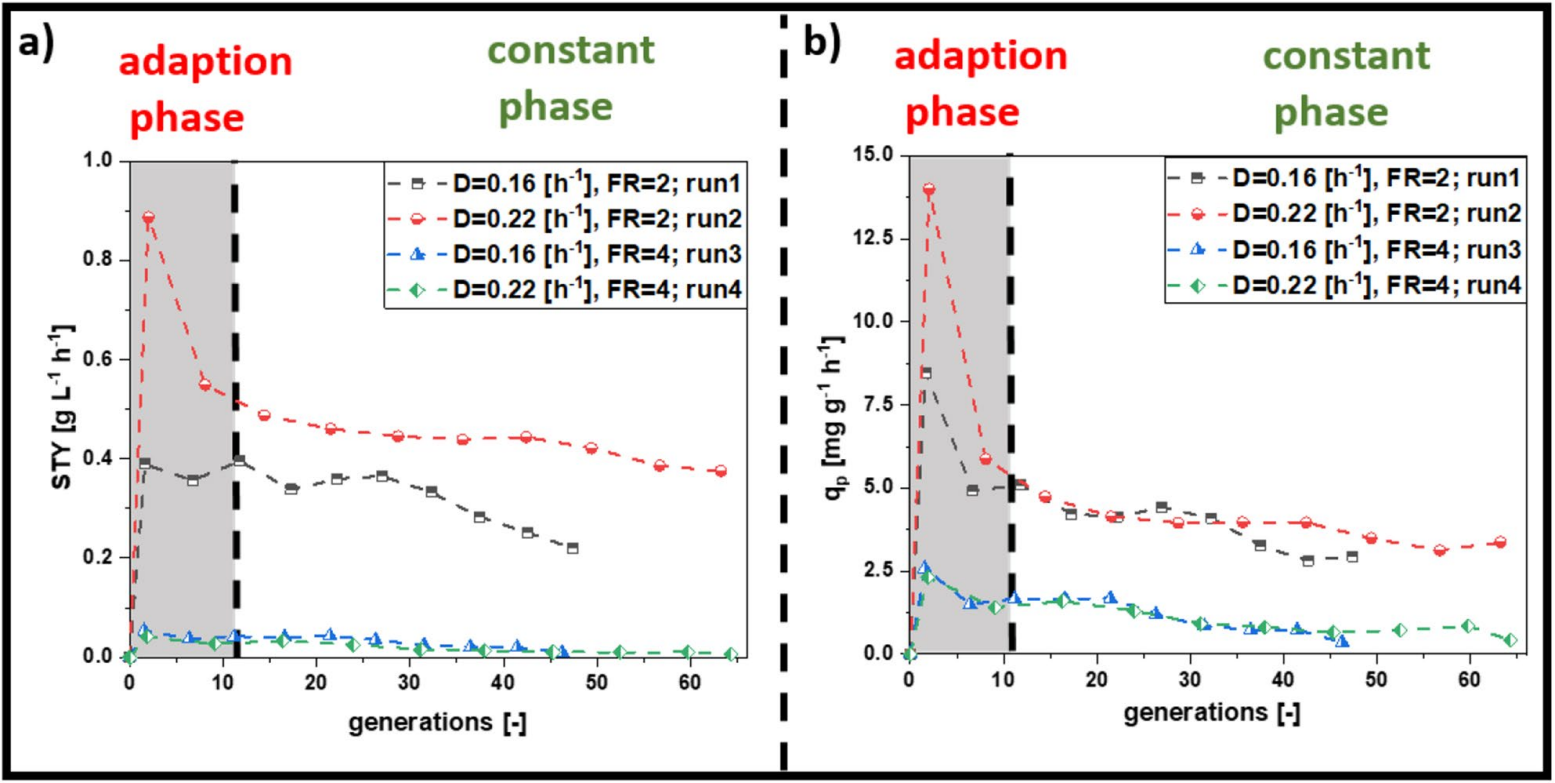
Results derived for cascaded continuous cultivation with mCherry, (**a**) showing the results for STY (space–time-yield) derived on a generation dependent manner, (**b**) showing q_p_ (specific productivity) on generation based behavior; high dilution rates and moderate feeding rates seem to be beneficial for production of mCherry (*FR* feed ratio, *D* dilution rate).

Assembling the gathered information allowed to set up a new screening strategy for mCherry, bearing a much lower experimental load, compared to the initial screening of Npro. The resulting constricted design space (Outcome of phase II, Fig. 5) was used to find suitable operation conditions for high STY and q_p_ in *E. coli* expressing mCherry (Fig. 6).

Although the adaptation phase for the mCherry strain was found shorter compared to the N-Pro strain (10 generations vs 20 generations, respectively), chosen process parameters had a comparable impact on specific productivity (Fig. 6b). Model terms for the reduced DoE can be found in Supplementary Data 3.2. Again, the process operated at D = 0.22/h with a feeding ratio = 2 resulted in a stable, long-time productivity for 180 h. The best process performance was found at dilution rate 0.22/h and a feed ratio of 2 (run 2, Fig. 6a), identical to N-Pro).

The proposed workflow (Figs. 4, 5) thus was appropriate to find well suited operating conditions for an uncharacterized recombinant BL21(DE3) strain and enabled stable long-time productivity with a highly reduced experimental load. Results indicate that the proposed workflow shows platform character, which can be used to ease the set-up of cascaded continuous cultivation for other uncharacterized strains.

### Comparison of cascaded continuous cultivations to fed-batch cultivations

**Figure 7:**
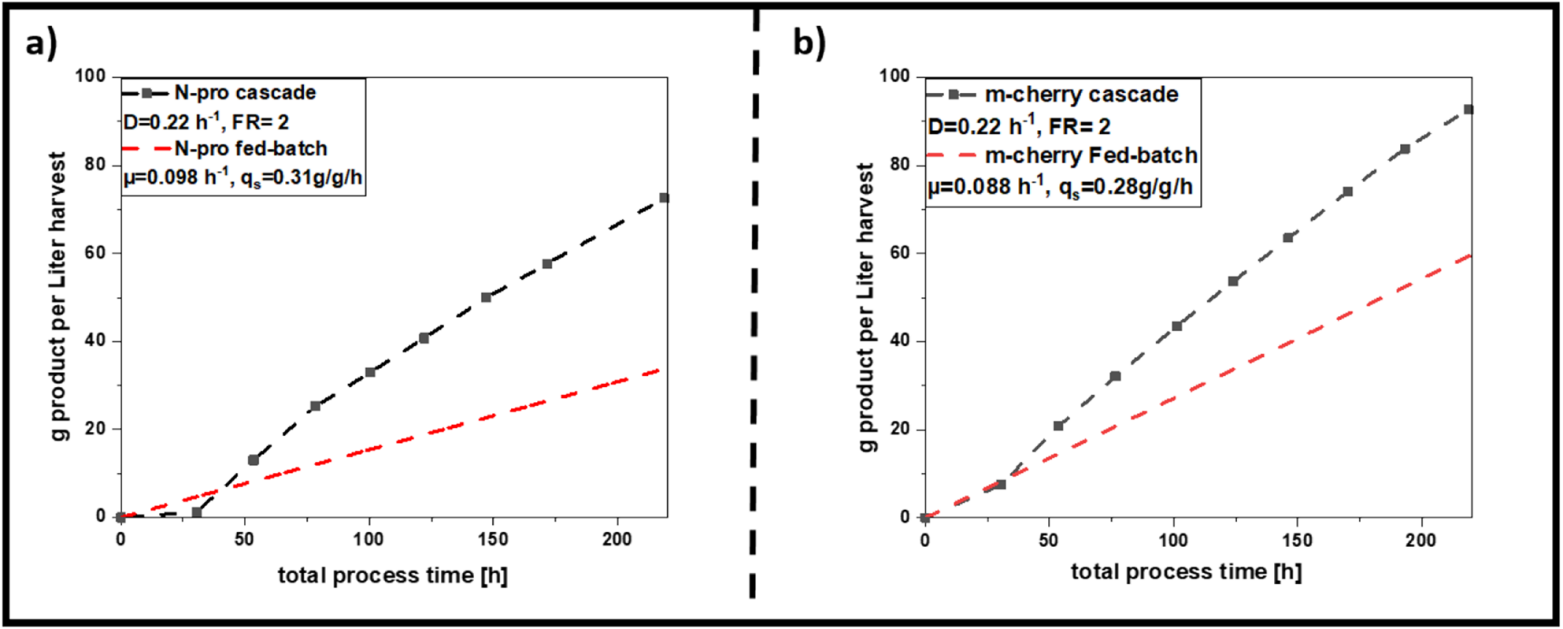
Space–time-yield calculated over the total process time for a fed batch and the cascaded continuous cultivation; For fed batches the maximum achieved titers were 5.09 g/L for the N-pro protein and 8.96 g/L for mCherry after each induction phase, CIP + SIP were assumed with 6 and 3 h respectively, batch phase with 6 h and non-induced fed-batch/continuous adaption phase with 12 h. Induction time for fed-batches was calculated with 10 h resulting in the dotted red lines, whereas cascaded continuous cultivation was calculated using cleaning and set-up times stated before, with the time-dependent results measured throughout induction phase given in the black curves for (**a**) N-pro and for (**b**) mCherry (*FR* feed ratio, *D* dilution rate, *CIP* clean-in-place, *SIP* sterilize-in-place).

We want to stress that the described process development for cascaded bio-manufacturing could be solely done with process engineering methods. This technique is thus ideal for being transferred to process development laboratories in industry. To strengthen this hypothesis STY from both processes were compared to state-of the art fed-batch cultivations which is the industrial golden standard. Total amount of produced protein was calculated for cascaded continuous process and fed-batch (Fig. 7a for N-pro and 7b for mCherry). As downtimes, such as CIP (clean-in-place), SIP (sterilize-in-place) and biomass cultivation times, can be reduced in cascaded processing, the total space time yield could be increased by a factor of 2.2 for N-pro protein cultivations () and 1.6-fold for mCherry STY.

## Discussion

Continuous cultivation with *E. coli* suffers from time-dependent productivity caused by the arising of non-producing subpopulations, which overgrow the producing population ^13,31,44^. Hence, common continuous cultivations with microbials bear mutation rates, plasmid loss and further genetic errors ^13,31,44^. The investigation of subpopulation evolution (i.e. via Flow cytometry analysis) was not within the scope of this study. In fact, for one of the chosen products such analytics would not have been technically feasible. In this study, we propose a workflow on how to set up continuous cultivations, keeping cells in an equilibrium state for induction times longer than 220 h using glycerol and lactose. Choosing recombinant protein titer (shown as q_p_ and STY) to determine cell equilibrium state in continuous cultivation, negligible long-time effects were observed at optimized process conditions. Results indicate low dilution rates (D = 0.1/h) to be unsuitable for achieving long-term stable productivity, independent from the applied uptake rates ^32,34^. We believe that high residence times (resulting from low dilution rates) enhance subpopulation diversification, as cells have more time to adapt to environmental conditions and to evolve a non-producing sub-population ^35,45,46^. High dilution rates (implementing low residence times) were found to result in stable productivity. The principle facilitates continuous bio-manufacturing, as higher volumetric rates would lead to higher amount of bleed and consequently higher STY. Even though cultivations at higher dilution rates, such as D = 0.24/h and D = 0.3/h were found to result in stable productivity (Fig. 3a,b, Supplementary Data 1.2), the feed ratio had a severe impact on the level of cell specific productivity. Careful optimizations in the feeding procedure (percentage of induction feed to feed) is thus necessary to achieve high and stable specific productivity.

As the ideal operating space for recombinant protein expression in cascaded continuous processing was intensively screened, obtained results were taken to propose a generic workflow for the establishment of cascaded continuous cultivation for *E. coli* BL21(DE3). We suggest to screen initial physiological parameters in a batch and two fed-batch runs (phase I) at first. Based on the initial screening experiments and the determination of physiological parameters (output of phase I) a reduced experimental design can be used to optimize process conditions in cascaded continuous cultivation (phase II). Testing the anticipated workflow showed that identical process parameters led to best results, independent from the recombinant protein. Stable specific productivity at highest space–time yield for the expression of both target proteins was found at a dilution rate of 0.22/h conducted at a feed ratio of 2 (q_p_ N-pro: 2.47 ± 13.3%; q_p_ mCherry = 3.97 ± 14.4%). Even tough ideal cultivations were well comparable for both proteins exploitation of different carbon sources and inducers could lead to different effects (i.e. altered inducer uptake rates ^18,42^).

Still, for new BL21(DE3) strains and products, we propose an experimental workflow with two phases using solely bioengineering methods. As parallelization of continuous processes can be used to speed up process development ^10^, we were able to establish stable continuous cultivations for a new target product within less than a month. Results presented in this study also show that cascaded continuous cultivation is superior to fed-batch cultivation for *E. coli* BL21(DE3) and hence is a suitable tool for bio-manufacturing. Consequently, the demonstrated experimental workflow could be used to set up and accelerate the implementation of a stable continuous upstream process for *E. coli*.

## Material and methods

### Strains and media

All performed cultivations were carried out using the *E. coli* strain BL21(DE3). The protein used for initial studies is an industrial protein (28.8 kDa), being linked to an N-pro fusion tag and integrated into a pET-30a plasmid system ^38^. The protein used for verification and transfer studies was mCherry, being integrated also in a pET30a plasmid system. Expression for mCherry was dominantly soluble in the cytoplasm, with small fractions being expressed as an inclusion body.

For all cultivations, a defined minimal medium referred to DeLisa et al. was used (Supplementary Data, Chapter 4.5). To cope for the antibiotic selection of pET30a systems, Kanamycin was supplemented to the medium resulting in a final concentration of 0.02 g/L^47^.

Preculture was assembled of 8.2 g/L glycerol while batch medium contained 20.4 g/L glycerol. The feed for biomass formation (reactor 1) contained 25 g/L of glycerol, while the mixed feed was altered according to the DoE approach (Fig. 1). Each step was conducted using the referred media, with altered sugar and trace-element concentrations adapted for the corresponding growth phases.

The preculture was carried out in Ultra yield flasks using 500 mL sterile DeLisa medium. Preculture was inoculated with 1.5 mL frozen bacterial stocks, stored at − 80 °C using 25% glycerol as antifreeze solution. They were cultivated over night at 37 °C, 230 rpm and a pH of 6.7 in an Infors HR Multitron shaker (Infors, Bottmingen Switzerland). The batch medium was inoculated the next day with the preculture using 20% of the reactor volume.

### Cascaded continuous cultivation

The used set up for the cascaded continuous cultivation is sketched in Fig. 1a. Biomass formation and protein expression were spatially separated, having sequentially operating chemostats implemented with separated feeds ^34^. In each run, one reactor was used for biomass formation and the derived biomass stream was transferred to the second reactor, controlled via pumps monitored by the process system PIMS Lucullus (SecureCell, Urdorf, Swiss). Biomass formation (reactor 1) was performed in a Labfors 3 bioreactor (max. wV: 2 L; Infors HT, Bottmingen, Switzerland). The reactor was coupled to a continuously operated stirred-tank reactor—(Minifors 2, max. wV: 1 L; Infors HT, Bottmingen, Switzerland), used for recombinant protein expression. Reactor 1 was aerated with a mixture of pressurized air and pure oxygen at 3 vvm and constantly stirred at 1000 rpm. Minifors reactors were aerated with 2 vvm and stirred at 1400 rpm. The off-gas concentrations of CO_2_ and O_2_ were monitored via BlueSens Gas sensors (BlueSens Gas analytics, Herten, Germany). Throughout all cultivations the dissolved oxygen (dO_2_) was kept above 40% by adjusting the ratio of pure oxygen and pressurized air. The dissolved oxygen was monitored with a fluorescence dissolved oxygen electrode Visiferm DO425 (Hamilton, Reno, NV, USA). As throughout the continuous phase the volume in the reactors was controlled with a dip pipe adjusted to the liquid surface, no stirrer cascade was implemented. pH was maintained at a constant value of 6.7 through all process phases and controlled by addition of NH_4_OH (12.5%). The pH was monitored with an EasyFerm electrode (Hamilton, Reno, NV, USA). In all three reactors, a batch was conducted with carbon concentrations of 20.4 g/L, yielding in 9–10 g dry cell weight of biomass per litre of cultivation broth. The end of the batch phase was determined by a drop in the CO_2_ signal.

Afterwards, the non-induced chemostat system was started by supplying the biomass reactor with a dilution rate of 0.14/h feed 1 (Table 1) and starting the transfer of biomass. For the cascaded continuous cultivation, the temperature in reactor 1 was set to 35 °C. To achieve an optimal protein expression the temperature in the second reactor was lowered to 31.5 °C for the N-pro protein ^22^, whereas 30 °C where applied throughout induction phase for mCherry, due to previous results obtained for soluble protein expression ^43^.

The so-called continuous adjustment phase and lasted over-night to achieve a steady state in all reactors, monitored by an equilibrium state of reactor 1 of derived pO_2_ and off gas signals. To initiate protein expression reactor 2 was supplied with the mixed feed containing glycerol and the inducer lactose, adjusted to the DoE approach. The volume throughput (Minifors 2, max. wV. 1 L) was adjusted to the desired dilution rate according to the design of experiment.

Experiments to determine uptake rates in mCherry, were performed in a Sartorius Biostat Cplus bioreactor (Sartorius, Göttingen, Germany) with 10 L working volume. Monitoring and control were performed via Lucullus (SecureCell, Urdorf, Swiss). The maximum glycerol uptake rate was determined in regularly sampling during the batch. Afterwards a lactose was added via pulse and uptake of solely lactose was evaluated. At a q_s,gly_ of about 0.15 g/g/h a static experiment was performed using a classic fed-forward approach and lactose pulses. Higher q_s,gly_ were screened using a linear ramp from about q_s,gly_ 0.25 to 0.55 g/g/h of the glycerol feed in combination with lactose pulses. While for BL21, uptake rates are similar ^42^, results might be different for other *E. coli* strains using lactose as inducing agent.

### Fed-batch cultivations

Pre-culture and process analytics were performed equivalent to the cascaded continuous cultivations. Fed-batches were conducted with an exponential feeding approach at a constant q_s,C_. Feed addition was started after the end of the batch, indicated by a drop of the CO_2_ signal. Feeding was conducted at q_s_ = 0.2 g/g/h and lasted over-night to generate 25–30 g/L biomass. Prior to induction temperature was adjusted to 31.5 °C for Npro and to 30 °C for mCherry. The exponential feeding was set to a q_s_ = 0.3 g/g/h for both proteins. Induction was performed via a 0.5 mM IPTG pulse to the fermentation broth and induction lasted for 10 h.

### Multivariate data assessment and process analytics

Multivariate data assessment of the performed experimental design was performed. Results were investigated for the statistical relevance of the model using the measure of fit (R^2^), the model predictability (Q^2^), the model validity and the model reproducibility, adjusted for degrees of freedom. Analysis of the process dataset was done by a multivariate data assessment (MODDE 12, Umetrics, Sweden). Time independent rates (space–time yield STY (mg/L/h) and specific productivity q_p_ (mg/g/h)) were chosen as responses to be evaluated for this study.

Process analytics were performed as described in previous studies ^18,22,32,40^ and further information can be found in Supplementary Sect. 4. Titer was determined using the described HPLC method ^48^. For quantification of the N-pro fusion protein, our industrial partner provided a purified standard reference. mCherry was quantified via a commercial available protein standard. Protein size of the expressed target proteins was determined via SDS-PAGE (results not shown). Time independent rates (specific productivity q_p_ (mg/g/h) and space–time yield STY (g/L/h) were chosen as model responses to be evaluated for this study. Rates were calculated as stated beneath:

$$q_{p} = \frac{{\frac{{c_{i} + c_{i - 1} }}{2}}}{{\frac{{X_{i} + X_{i - 1} }}{2} \times (t_{i} - t_{i - 1} )}}.$$

q_p_: specific productivity [mg/g/h], $c_{{\text{i}}}$: product concentration of sample i [mg/L], $X_{{\text{i}}}$: biomass concentration of sample i [g/L], $t_{{\text{i}}}$: cultivation time at timepoint of sample i [mg/L].

Space–time-yield used for comparison, was calculated by the following Eqs. (2) and (3):

2 $$STY = \frac{{\frac{{c_{i} + c_{i - 1} }}{2} \times \Delta {\dot{\text{F}}}_{{\text{i}}} }}{{V_{R} }},$$

3 $${\text{with}}\; \Delta {\dot{\text{F}}}_{{\text{i}}} = {\dot{\text{F}}}_{{\text{i}}} - {\dot{\text{F}}}_{{{\text{i}} - 1}} = \Delta \frac{{{\dot{\text{X}}}_{{\text{i}}} }}{2} + { }\Delta {\dot{\text{F}}}_{ind. Feed} + { }\Delta {\dot{\text{F}}}_{Base} .$$

STY: space–time-yield [g/L/h], $c_{{\text{i}}}$: product concentration of sample i [g/L], $F_{{\text{i}}}$: volumetric flow through recombinant protein producing reactor at timepoint of sample i [L/h], $\dot{X}_{{\text{i}}}$: biomass flux derived from reactor 1 at timepoint of sample i [L/h], ${\dot{\text{F}}}_{ind. Feed}$: volumetric rate of inducer feed in reactor 2 [L/h] (comp. to Fig. 1a), ${\dot{\text{F}}}_{Base}$: volumetric rate of base in reactor 2 [L/h] (comp. to Fig. 1a).

## Supplementary Information

Supplementary Information.
