# Supplementary material for: Cascaded processing enables continuous upstream processing with E. coli BL21(DE3)
Source: Sci Rep. 2021 Jun 1;11:11477. doi: 10.1038/s41598-021-90899-9 (PMC8169658; doi:10.1038/s41598-021-90899-9)
Supplement: Supplementary file 1 — Supplementary Information. [file 41598_2021_90899_MOESM1_ESM.pdf]

Supplementary material

for

Cascaded processing enables continuous Upstream Processing with *E. coli* BL21(DE3)

Stefan Kittler[1], Christoph Slouka[1], Andreas Pell[1], Roman Lamplot[1], Mihail Besleaga[1], Sarah Ablasser[1], Christoph Herwig[2], Oliver Spadiut and Julian Kopp[1,§]

[1]*Research Division Integrated Bioprocess Development, Institute of Chemical, Environmental and Bioscience Engineering, TU Wien Vienna, Austria*

[2]*Institute of Chemical, Environmental and Bioscience Engineering, TU Wien, Getreidemarkt 9/166, 1060, Vienna, Austria*

[§]Correspondence and Requests for materials should be addressed to:

Julian Kopp, TU Wien, Institute of Chemical, Environmental and Bioscience Engineering, Research Division Integrated Bioprocess Development, Gumpendorfer Straße 1a, 1060 Vienna, Austria. Tel.: +43 1 58801 166485, Email: julian.kopp@tuwien.ac.at

1. Pre-experiments conducted to find out parameters for the full factorial design conducted with the N-pro fusion protein

1.1 Investigation to find appropriate feeding rates

The amount of fed carbon sources, was found to have a severe influence throughout fed-batches onto recombinant protein formation. To test the influence in continuous cultivation different ratios of biomass and inducer feed at a set dilution rate of 0.15 h^{-1} were tested for the N-pro fusion protein. Thereby we varied the ratio of the inducer feed and fed biomass from the second reactor: Initial ratios ($\dot{F}_{biomass}$ to $\dot{F}_{indFeed}$) of 9:1 ($q_{s\,C}$ = 0.11 g/g/h) and 8:2 ($q_{s,C}$ = 0.23 g/g/h) were found to result in time-dependent decrease of productivity whereas a ratio of 7:3 ($q_{s,C}$ = 0.42 g/g/h) resulted in a higher and stable productivity (Figure S1). This is most likely because (i) higher biomass concentrations were achieved resulting in an overall higher titer and (ii) enough energy was provided, so that cells could maintain intracellular energy levels for recombinant protein production.

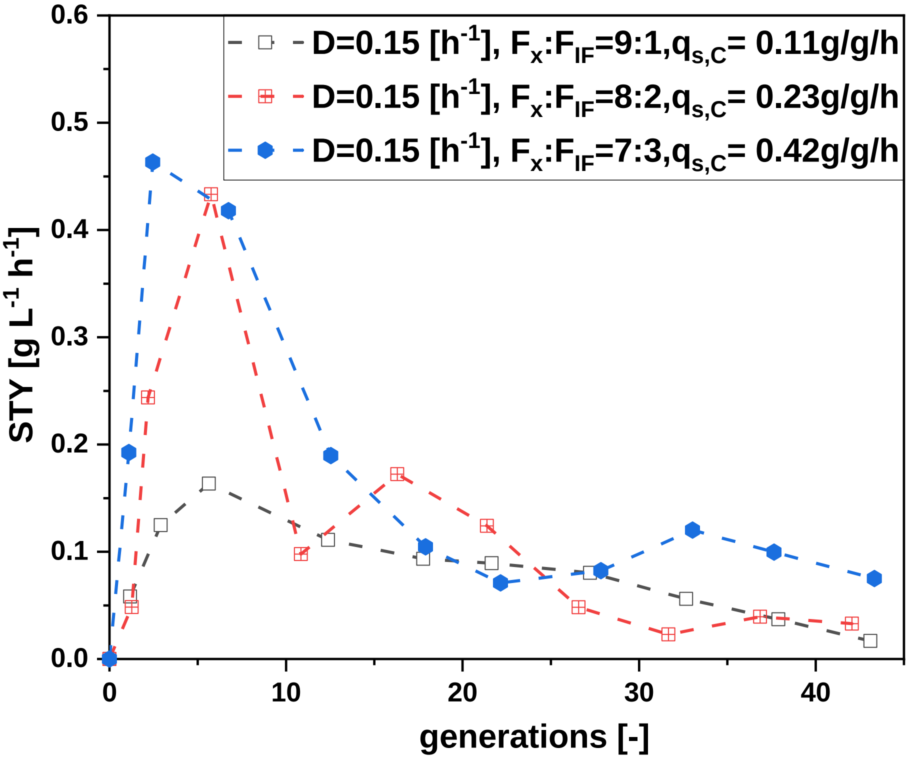

Supplementary Figure 1: Pre-experiments, showing a dependency of the applied ratio between biomass distribution of seed reactor and the amount of fed inducer feed, ratios of 9:1 and 8:2 were found to be

inefficient whereas a ratio of 7:3 was found to yield in long stable productivity, higher ratios were ruled out to avoid high levels of sugar accumulation. (Fx = biomass flow [mL h-1], FIF = inducer feed flow [mL h-1], D = dilution rate, STY = space-time-yield)

1.2. Investigation of dilution rates close to μ_{max}

In previous studies higher dilution rates were found to be beneficial for stable protein production. The effects on how close to μ_{max} or D_{crit} a process layout can potentially be conducted, still needs to be clarified. Therefore, we tested, whether higher dilution rates can also be applicable. We performed a run at the determined feed ratio of 2 of the DoE designs, at a dilution rate of 0.3 h^{-1} (using the determined 7:3 ratio), close to the μ_{max} (at 0.38 h^{-1}). Results indicated that the run was compared to the pre-experiments (supplementary data, section 1.1., Figure S1), insufficient regarding q_p and STY (Figure S2). Higher dilution rates were thought to imply higher STY, which was not seen throughout this experiment. Nevertheless, it was highly interesting to monitor, no shifts in productivity were monitored when applying higher dilution rates. Where an adaption phase in the range of 10-20 generations was seen for first experiments conducted with the Npro fusion protein, throughout this experiment adaption phase lasted for 10 generations only. We hypothesize, that cells can be maintained in their metabolic state by applying higher dilution rates as cells do not suffer from evolutionary tribes, due to decreased residence times

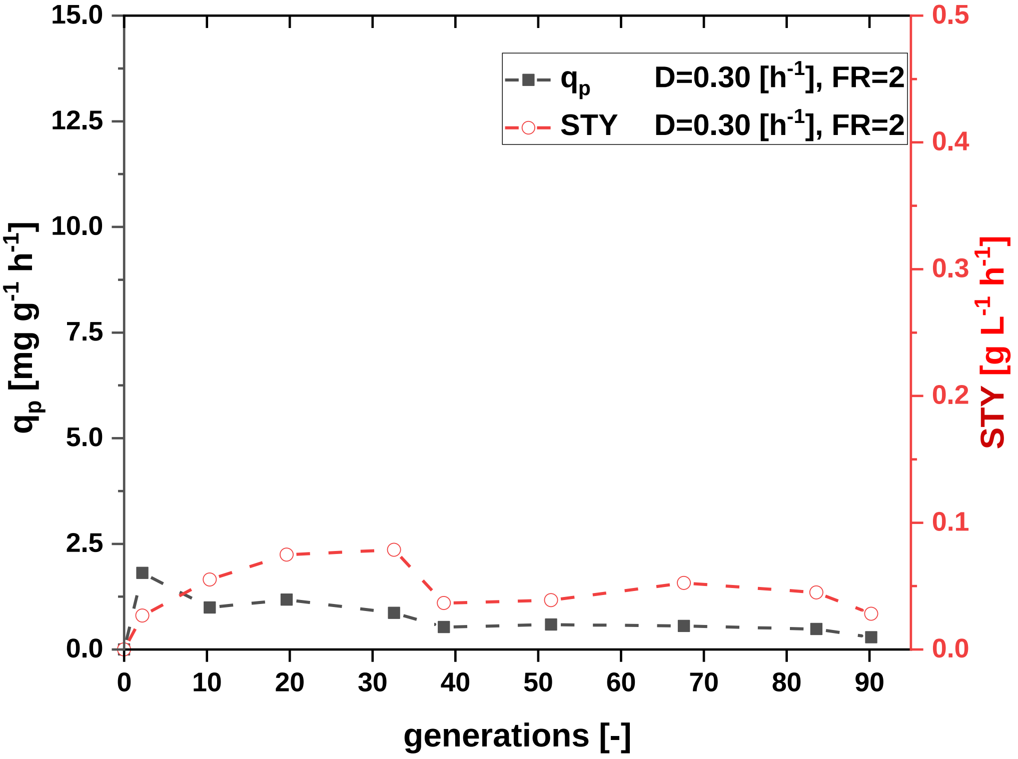

Supplementary Figure 2: Pre-experiments, showing the dependency onto q_p (specific productivity) and STY (space-time-yield) for the applied dilution rate of 0.3; even tough productivity was found to be at very low levels, cells never stopped producing indicating the trend that cells can be kept in their metabolic state when applying higher dilution rates in order to avoid evolutionary tribes. (D = dilution rate, FR = feed ratio)

2. Additional Data for experiments conducted with the N-pro fusion protein

2.1. Summary of process parameters

Table S1 summarizes the process parameters and responses found out throughout the study conducted for the design of experiment, whereas Table S2 summarizes process parameters and results for the optimization study conducted for the N-pro fusion protein.

Supplementary Table 1: Comparison of all runs according to the experimental design space shown in paper Figure 2. All values are calculated as an average after the monitored decrease of productivity in the adaption phase (20 generations) to evaluate the process regarding the long-term productivity. (q_p = specific productivity, STY = space-time-yield)

Run	dilution rate [h^{-1}]	$q_{s,gly}$ [$g\ g^{-1}\ h^{-1}$]	$q_{s,lac}$ [$g\ g^{-1}\ h^{-1}$]	$c_{glycerol}$ [$g\ L^{-1}$]	$c_{lactose}$ [$g\ L^{-1}$]	ratio c_{gly}/c_{lac}	q_p [$mg\ g^{-1}\ h^{-1}$]	STY [$mg\ L^{-1}\ h^{-1}$]
**1**	0.10 ± 0	0.40 ± 0.09	0.07 ± 0.02	400	100	4	0.21 ± 0.87	17.1 ± 3.9
**2**	0.20 ± 0.01	0.17 ± 0.05	0.30 ± 0.09	100	200	0.5	0.81 ± 0.37	108.5 ± 46.3
**3**	0.19 ± 0.02	0.40 ± 0.03	0.10 ± 0.01	400	100	4	1.21 ± 0.40	216.9 ± 52.7
**4**	0.10 ± 0.01	0.07 ± 0.01	0.14 ± 0.01	100	200	0.5	0.12 ± 0.04	11.1 ± 3.1
**CP**	0.15 ± 0.01	0.31 ± 0.11	0.12 ± 0.05	220	110	2	0.54 ± 0.16	52.3 ± 15.6

Supplementary Table 2: Comparison of all runs performed for the optimization of the N-pro protein (compare to paper Figure 3). All values are calculated as an average after the monitored adaption phase (20 generations) to evaluate the process regarding the long-term productivity. (q_p = specific productivity, STY = space-time-yield)

Run	dilution rate [h^{-1}]	$q_{s,gly}$ [$g\ g^{-1}\ h^{-1}$]	$q_{s,lac}$ [$g\ g^{-1}\ h^{-1}$]	$c_{glycerol}$ [$g\ L^{-1}$]	$c_{lactose}$ [$g\ L^{-1}$]	ratio c_{gly}/c_{lac}	q_p [$mg\ g^{-1}\ h^{-1}$]	STY [$mg\ L^{-1}\ h^{-1}$]
**SP1**	0.22 ± 0.00	0.48 ± 0.02	0.23 ± 0.02	220	110	2	2.47 ± 0.33	340.5 ± 22.6
**SP2**	0.15 ± 0.02	0.46 ± 0.19	0.14 ± 0.11	472	100	4.72	0.04 ± 0.04	7.2 ± 8.2

**High D**	0.24 ± 0.01	0.37 ± 0.03	0.18 ± 0.01	220	110	2	0.58 ± 0.58	112.4 ± 106.0

2.2. Statistical output for the full-factorial DoE derived for the N-pro fusion protein

A common full factorial design using a CCC (central composite circumscribed) approach was conducted. Thereby the dilution rate and the feed ratio were varied according to the design of experiment evaluating specific productivity (q_p [mg g-1 h-1]) and space time yield (STY [mg L-1 h-1]). In a full factorial designs only linear and interaction terms can be investigated. Star-point experiments help to determine the effects of quadratic interactions of the studied factors investigated in Figure 3 (main manuscript).

2.2.1. Effects onto response space time yield (mg/L/h)

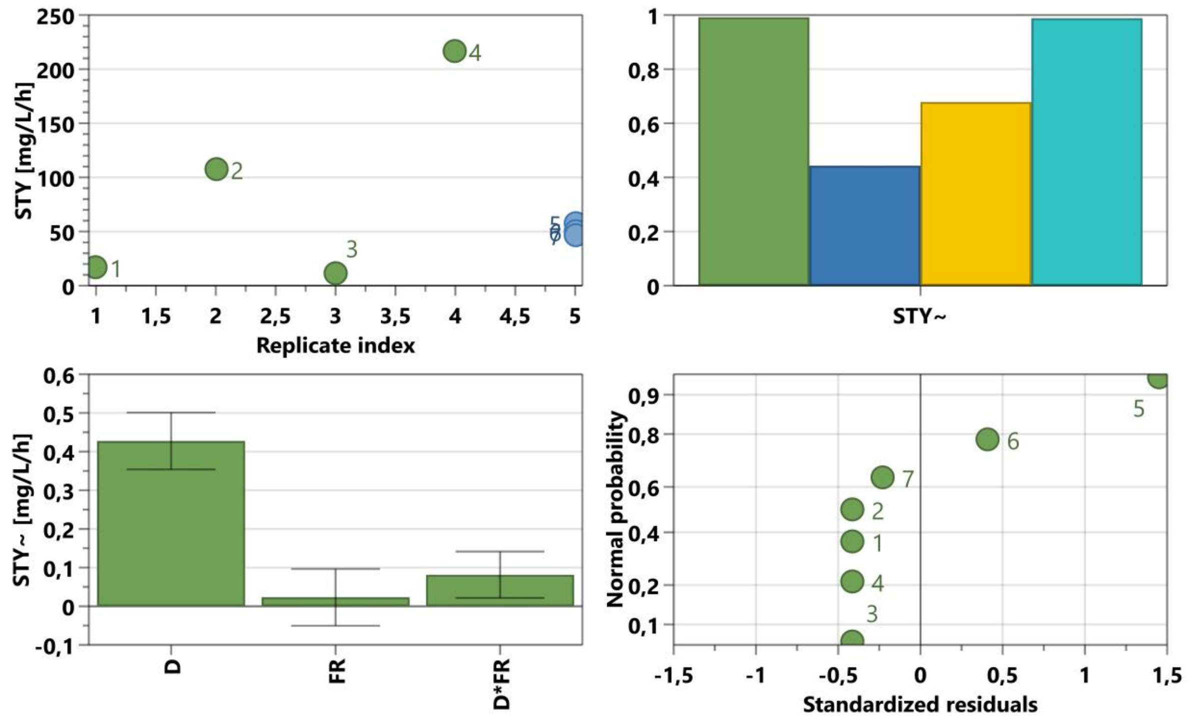

Supplementary Figure 3: Summary of the fit for the response STY (space-time-yield), showing the replicates at a non-transformed level, the summary of fit indicating a high R^2 and sufficient Q^2, a high model validity and high reproducibility;. The coefficient plot indicates that dilution rate and the interaction term of dilution rate and feed ratio have a significant positive influence onto the STY. Feed ratio also has a positive impact, but is only included in the model as this is a hierarchical model description. A high increase in dilution rate leads to wash out and decreasing productivity as shown in

section 1.2. High dependency of the applied volumetric rate can be confirmed for the STY by the statistical analysis; furthermore, the residual normal probability is given for STY. (D = dilution rate, FR = feed ratio)

2.2.2. Effects onto response specific productivity (q_p = mg/g/h)

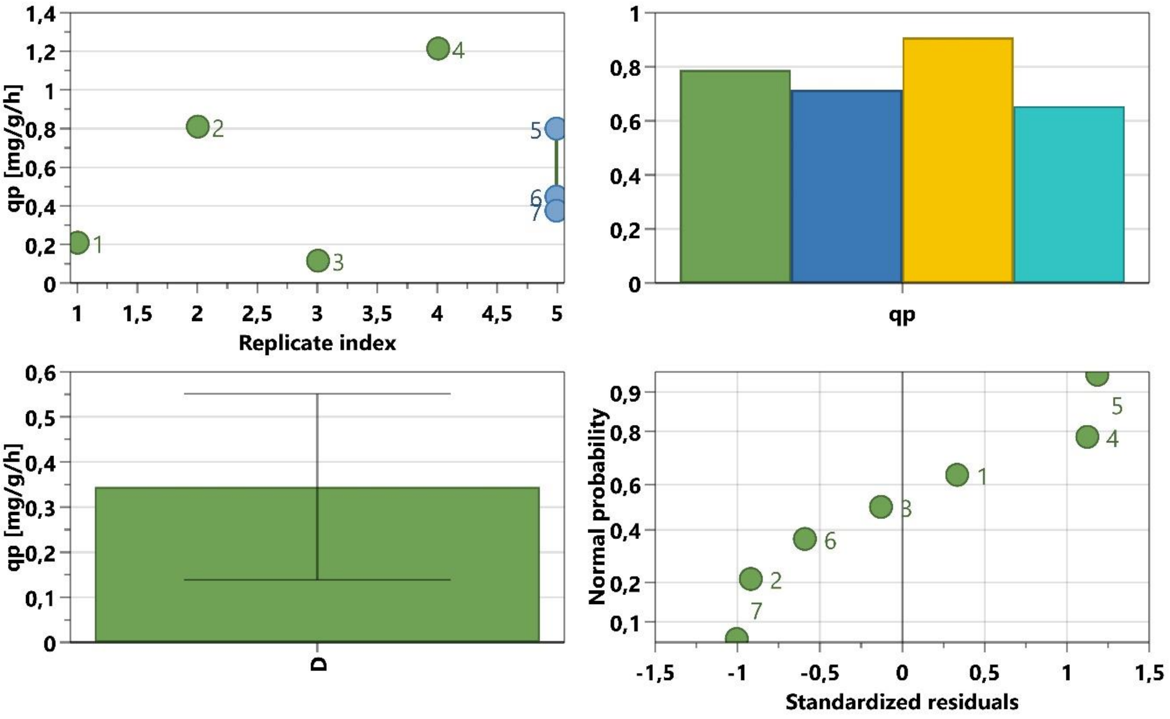

Supplementary Figure 4: Summary of the fit for the response q_p (specific productivity), showing the replicates at a non-transformed level. Summary of fit is indicating a good R^2 to Q^2 ratio, a high model validity and high reproducibility, nevertheless received measure of fit and reproducibility for q_p is smaller when compared to R^2 for the response STY (space-time-yield). This might be as dilution rate has a higher impact onto process performance regarding STY, when compared to q_p (dilution rate and space time yield are both dependent on volumetric rates) coefficient plot indicates that dilution rate, has a highly significant influence, whereas the applied feed ratio also has a positive influence, however the term is not significant for specific productivity and thus was excluded; furthermore, the residual normal probability is given for q_p. (D = dilution rate)

3. Additional Data for experiments conducted with m-cherry

3.1. Summary of process parameters

Supplementary Table 3: Comparison of all runs according to the experimental design space shown in paper Figure 5 for m-cherry. All values are calculated as an average after the monitored decrease of productivity in the adaption phase (10 generations) to evaluate the process regarding the long-term productivity. (q_p = specific productivity, STY = space-time-yield)

Run	dilution rate [h^{-1}]	$q_{s,gly}$ [$g\ g^{-1}\ h^{-1}$]	$q_{s,lac}$ [$g\ g^{-1}\ h^{-1}$]	$c_{glycerol}$ [$g\ L^{-1}$]	$c_{lactose}$ [$g\ L^{-1}$]	ratio c_{gly}/c_{lac}	q_p [$mg\ g^{-1}\ h^{-1}$]	STY [$mg\ L^{-1}\ h^{-1}$]
**1**	0.15 ± 0.02	0.39 ± 0.02	0.20 ± 0.01	220	110	2	3.87 ± 0.74	318.3 ± 56.8
**2**	0.21 ± 0.01	0.52 ± 0.02	0.26 ± 0.01	220	110	2	3.97 ± 0.57	439.4 ± 46.5
**3**	0.15 ± 0.01	0.70 ± 0.06	0.18 ± 0.01	400	100	4	1.03 ± 0.47	28.0 ± 11.3
**4**	0.22 ± 0.00	0.82 ± 0.11	0.28 ± 0.04	400	100	4	0.91 ± 0.35	17.2 ± 8.2

3.2. Statistical output for the full-factorial DoE derived for the m-cherry protein

3.2.1. Effects onto response space time yield (g/L/h)

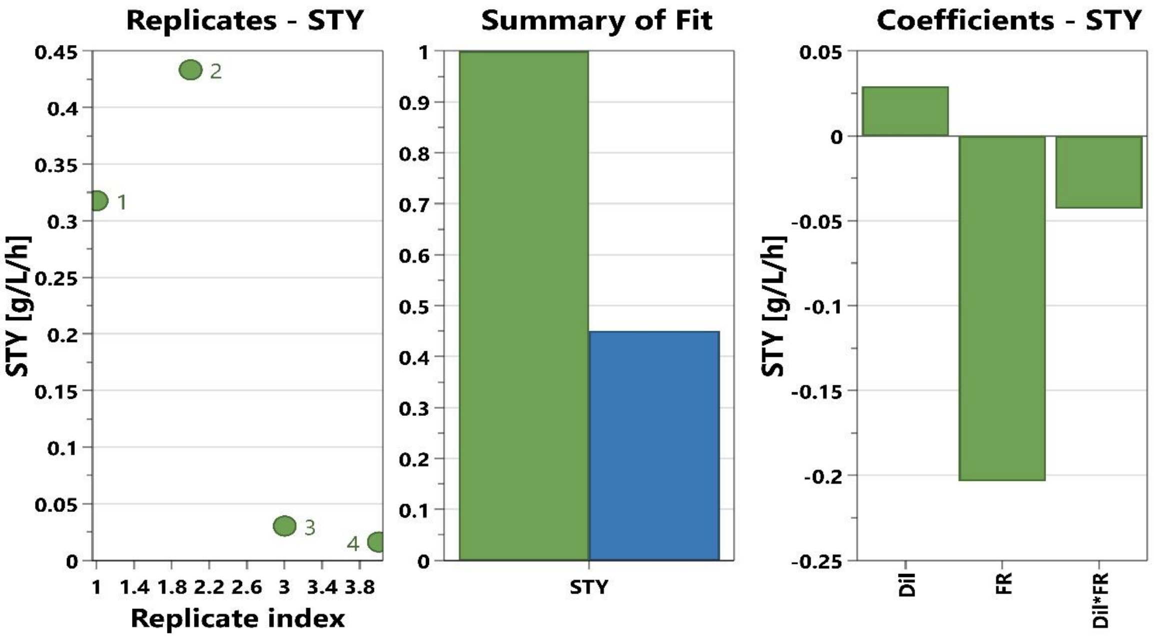

Supplementary Figure 5: Summary of the fit for the response STY (space-time-yield), for m-cherry, as centerpoints have not been conducted in the reduced DoE, the summary of fit lacks in model validity and replication terms. Feed ratio was found to have a severe influence onto STY, showing that lower feeding ratios tend to have much better results; as previously determined optimum dilution rates were used dilution rate showed only a meager impact. (Dil = dilution rate, FR = feed ratio)

3.2.2. Effects onto response specific productivity (q_p = mg/g/h)

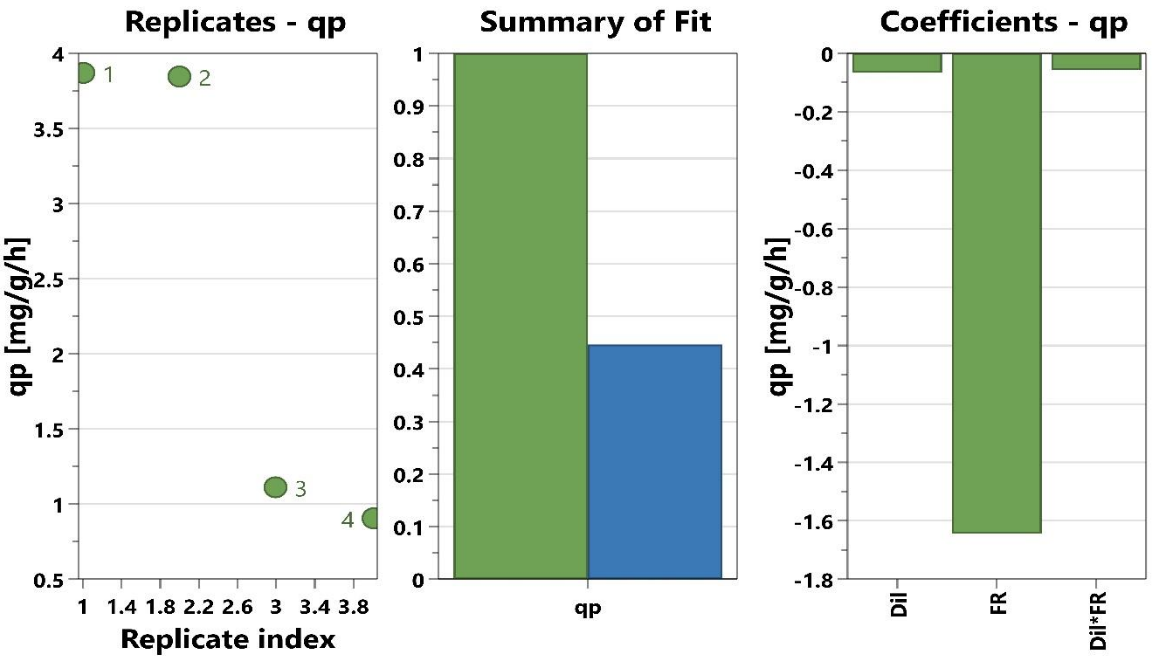

Supplementary Figure 6: Summary of the fit for the response q_p (specific productivity), for m-cherry, as center points have not been conducted in the reduced DoE, the summary of fit lacks in model validity and replication terms. Feed ratio was found to have a severe influence onto q_p, showing that lower feeding ratios tend to have much better results; as previously determined optimum dilution rates were used dilution rate showed only a meager impact. (Dil = dilution rate, FR = feed ratio)

4. Material und Methods

4.1. Process analytics

Before the start of induction phase a sample was taken and further samples of each reactor were taken on a daily manner during the induced process phase. For each sample, the biomass was measured optically at 600 nm (OD_{600}) as well as gravimetrically by weighing dry cell weight (DCW). For the OD_{600} measurements with a Genesys 20 photometer (Thermo Scientific, Waltham, MA, USA), the samples were diluted with deionized water to keep them within the linear range of the photometer (0.2 – 0.8 AU). The dry cell weight was determined in triplicates, by pipetting 1 mL of the homogenous cell solution into pre-dried and -weighted 2 mL Eppendorf tubes, (Eppendorf, Hamburg, Germany). The tubes were centrifuged at 14000 rpm for 10 min at 4 °C. The supernatant was stored at -20 °C in 2 mL Eppendorf tubes for HPLC measurements to determine sugar concentrations in the broth. Subsequently, the pellets were resuspended in 1 mL of 0.9% NaCl solution and then centrifuged with the same parameters. After this washing step the supernatant was discarded, and the pellets were dried for at least 72 h at 105 °C and then weighted.

In addition, a sample for flow cytometry (FCM) was taken to determine cell viability. The broth was diluted 1:100 with 0.9 % filtered NaCl solution and stored at 4 °C until it was measured on the same or next day. The measurements were performed using a Cube 8 (Sysmex, Görlitz, Germany) and the data analysis with the program FCS Express (De Novo Software, Pasadena, USA). The FCM samples were diluted to an OD_{600} of approximately 0.01 using a 0.9 % NaCl solution and then measured according to the described procedure in Langemann *et al.*. To determine the viability, the cells were dyed using DiBAC4(3) (bis-(1,3-dibutylbarbituricacid-trimethineoxonol) and Rh414 dye. DiBAC4 is a membrane potential-sensitive fluorescent dye, while Rh414 binds to the membrane and enables to exclude non cellular background [1]. As signals of Rh414 were not visible due to auto-fluorescence of m-cherry, only DiBAC4(3) was added to flow cytometry analysis of m-cherry samples.

Furthermore, product samples were taken throughout induction phase. 10 mL of cell broth was pipetted in a 50 mL falcon tube and centrifuged for 10 – 15 min at 4 °C and 9500 rpm. The supernatant was discarded, and the pellet was stored at -20 °C.

To test for sugar accumulation throughout the process, fermentation supernatants were measured via anion-exchange-HPLC (Thermo Scientific, Waltham, MA, USA) using a Supelcogel-column with an eluent of 0.1% H_3PO_4 and a flow of 0.5 mL/min. Standards had a

concentration of 0.5, 1, 5, 10, 25 and 50 g L^{-1} of every sugar used throughout all fermentations. The HPLC run lasted for 30 min and chromatograms were analysed using a Chromeleon Software (Dionex, Sunnyvale, CA, USA). Accumulation of lactose, galactose, glycerol and acetate were monitored in order to calculate real specific feeding- and accumulation rates.

4.2. Product titer analytics

The product samples were taken as described in chapter 2.4. Pellets were resuspended in 40 mL of Lysis buffer (0.1 M TRIS, 10 mM EDTA, pH = 7.4) using an Ultraturrax device (T 10 basic ULTRA-TURRAX®, Staufen, Germany). Afterwards, the homogenous sample was homogenized using a GEA Pandsa Homogenizer (Düsseldorf, Germany) at 1300 bar for 4 passages (2 min). The disrupted cells were then centrifuged at 9800 rpm at 4 °C for 10 min. Subsequently, the supernatant was discarded, and the pellet was washed with 30 mL deionized water and again centrifuged with the same parameters. Afterwards the supernatant was discarded again, and the pellet was resuspended in 10 mL dH_2O and aliquoted into ten separate 2 mL Eppendorf tubes á 1 mL. They were centrifuged for 20 min at 4 °C and 14 000 rpm. The supernatant was removed, and the pellet was frozen at – 20°C. The protein concentration was determined via HPLC (Thermo Scientific, Waltham, MA, USA). The IB was dissolved in 1 mL solubilisation buffer (7.5 M Guanidine Hydrochloride, 62 mM Tris at pH = 8) and then filtered using 0.2 µm RCF filters. The target protein was quantified via reversed phase HPLC using a polyphenyl column (Waters, USA). The eluent was a mixture of water with 0.1% TFA (trifluoroacetic acid) and acetonitrile with 0.1% TFA with a flow of 1.2 mL*min $^{-1}$, which was changed with a gradient. Analytic runs lasted for 10.1 min. Standard concentrations were 50, 140, 225, 320, 500,1000 and 2000 mg m L^{-1} of an industrial supplied reference. For m-cherry same calibration was conducted with BSA using same concentrations as stated before.

4.3. Calculation of dilution rate

The dilution rate was determined with monitored feed volumes over time, while the reactor volume was kept constant. Via the monitored weight we were able to calculate the real dilution rate.

$$D_{Ind.\ Reactor} = \frac{\dot{F}_{indFeed} + \dot{F}_{biomass}}{V_{reactor}}$$

$D_{Ind.\ Reactor}$ ...	dilution rate in the reactor for protein expression [h^{-1}]
$F_{indFeed}$ ...	applied feeding rate of the induction feed [$mL\ h^{-1}$]
$F_{biomass}$ ...	applied biomass flow to the protein expression reactor [$mL\ h^{-1}$]
$V_{reactor}$ ...	volume in the protein expression reactor [L]

4.4. Calculation of specific substrate uptake rate $q_{s,c}$

The specific substrate uptake rate was calculated according to the following equation. Real sugar uptakes were used, evaluated by the accumulated component subtracted from the fed substrate.

$$q_{s,c} = \frac{c_{c,Feed} * V_{Feed,in} - c_{c,acc} * V_{reactor}}{\Delta t * X_{\Delta t}}$$

$q_{s,c}$ ...	specific uptake rate of the corresponding component [$g\ g^{-1}\ h^{-1}$]
$c_{c,Feed}$ ...	concentration of the corresponding component in the feed [$g\ L^{-1}$]
$V_{Feed,in}$ ...	feed volume into the reactor in the timespan Δt [L]
$c_{c,acc}$ ...	concentration of the corresponding component which accumulated in the reactor (HPLC measurement) [$g\ L^{-1}$]
$V_{reactor}$ ...	volume in the protein expression reactor [L]
Δt ...	timespan in which the uptake rate is calculated [h]
$X_{\Delta t}$ ...	average biomass in the protein expression reactor during the timespan Δt [g]

4.5. Media composition

Supplementary Table 4: Composition of the defined medium according to DeLisa et al., which was used for all performed cultivations [2]. Trace elements were prepared in separate stocks and added after sterilization of main media components.

DeLisa Medium	final conc. [g/l]
Glycerol	22,00
KH_2PO_4	13,30
$(NH_4)_2HPO_4$	4,00
Citric acid	1,70

Trace-elements (prepared separately)	final conc. [g/l]
$MgSO_4 * 7\ H_2O$	1,20
Fe(III) citrate	0,1000
EDTA	0,0084
$Zn(CH_3COO)_2 * 2\ H_2O$	0,0130
$CoCl_2 * 6\ H_2O$	0,0025
$MnCl_2 * 4\ H_2O$	0,0150
$CuCl_2 * 2\ H_2O$	0,0012
H_3BO_3	0,0030
$Na_2MoO_4 * 2\ H_2O$	0,0025
Thiamine HCl	0,0045

References supplementary section

1 Langemann, T., Mayr, U. B., Meitz, A., Lubitz, W. & Herwig, C. Multi-parameter flow cytometry as a process analytical technology (PAT) approach for the assessment of bacterial ghost production. *Applied Microbiology and Biotechnology* **100**, 409-418, doi:10.1007/s00253-015-7089-9 (2016).

2 DeLisa, M. P., Li, J., Rao, G., Weigand, W. A. & Bentley, W. E. Monitoring GFP-operon fusion protein expression during high cell density cultivation of Escherichia coli using an on-line optical sensor. *Biotechnol Bioeng* **65**, 54-64 (1999).
